# S-100B Protein and Chronic Subdural Hematoma

**DOI:** 10.3389/fneur.2013.00024

**Published:** 2013-03-15

**Authors:** Miguel Gelabert-González, Eduardo Aran-Echabe, Ramón Serramito-García

**Affiliations:** ^1^Department of Surgery, University of Santiago de CompostelaSantiago de Compostela, Spain

We are surprised by the conclusions of a recent paper by Persson et al. (2012), “Case report: extreme levels of serum S-100B in a patient with chronic subdural hematoma,” which suggest that S-100B protein is an important marker for chronic subdural hematoma (CSDH). In our opinion, and that of other authors, S-100B is a significant marker of multiple neurological pathologies.

Chronic subdural hematoma is a relatively common complication, especially among the elderly, where the incidence is estimated as 7.4/100,000. Two circumstances account for its high incidence among the elderly; the extensive brain atrophy often found in the elderly or alcoholics, and long-term use anticoagulant (Gelabert et al., 2001). CSDH in patients less than 50 years old is rare, and when it does occur it usually points to a predisposing factor, as in this case, where a brain metastasis led to the formation of a hematoma (Gelabert-González et al., 2012).

Moreover, while the radiological image of the patient in their study shows a hematoma, it is of small and therefore leads one to question how this can be responsible for such a midline shift. This suggests that something more must be involved, as was revealed at autopsy where a metastases was identified.

In a recent paper, Kruijff and Hoekstra (2012) state that the protein S-100B is probably the best biomarker for melanoma, having potential to identify high-risk stage III melanoma patients who may benefit from adjuvant systematic treatment. Since an effective (adjuvant) therapy for loco-regional metastatic and disseminated melanoma has only been recently introduced, the diagnostic of S-100B, they argue, is set to increase in the near future.

It therefore appears inappropriate to consider differential levels of S-100B as indicative of the evolution of CSH, as suggested by the paper's title, since they can be more indicative of a melanoma and its metastasis.
